# Perceptions, attitudes, and willingness of the public in low- and middle-income countries of the Arab region to participate in biobank research

**DOI:** 10.1186/s12910-022-00855-z

**Published:** 2022-12-01

**Authors:** Mamoun Ahram, Fatma Abdelgawad, Samar Abd ElHafeez, Ahmed Samir Abdelhafiz, Maha Emad Ibrahim, Alya Elgamri, Zeinab Mohammed, Karima El-Rhazi, Eman Elsebaie, Ehsan Gamel, Manal Shahouri, Nada Taha Mostafa, Latifa Adarmouch, Henry Silverman

**Affiliations:** 1 School of Medicine, The University of Jordan, Amman, Jordan; 2 Faculty of Dentistry, Cairo University, Cairo, Egypt; 3 High Institute of Public Health, Alexandria University, Alexandria, Egypt; 4 National Cancer Institute, Cairo University, Cairo, Egypt; 5 Faculty of Medicine, Suez Canal University, Ismailia, Egypt; 6 Faculty of Dentistry, University of Khartoum, Cairo, Egypt; 7 Faculty of Medicine, Beni-Suef University, Beni Suef, Egypt; 8 Faculty of Medicine of Fez, Sidi Mohamed Ben Abellah University, Fez, Morocco; 9 Faculty of Medicine, Cairo University, Cairo, Egypt; 10 Faculty of Dentistry, University of Khartoum, Khartoum, Sudan; 11 Amman, Jordan; 12 Cairo, Egypt; 13 Faculty of Medicine, Cadi Ayyad University, Marrakesh, Morocco; 14 University of Maryland School of Medicine, Baltimore, MD USA

**Keywords:** Biobanks, Arab countries, Public, Perceptions, Attitudes, Data sharing, Genomic research, Return of individual research results, Trust, Privacy

## Abstract

Population-based genomics studies have proven successful in identifying genetic variants associated with diseases. High-quality biospecimens linked with informative health data from diverse segments of the population have made such research possible. However, the success of biobank research depends on the willingness of the public to participate in this type of research. We aimed to explore the factors associated with the willingness of the public to participate in biobank research from four low- and middle-income countries in the Arab region (Egypt, Jordan, Morocco, and Sudan). We used a previously validated questionnaire to assess several constructs that included the public’s perceptions, attitudes, and willingness to participate in biobank research. We recruited 967 participants. More than half did not have prior awareness of biobanks. Participants’ willingness to donate biospecimens and health data was less than 10%. Our results also showed that participants harbored concerns with trust, privacy, and with data-sharing involving international researchers. Predictors of willingness to participate in biobank research included no previous involvement in research and positive attitudes toward biobanks. Finally, our study showed several differences between the four countries regarding several of the investigated constructs. We conclude there should be additional efforts to raise public awareness and enhance perceptions of the public in biobanking research to enhance trust. We further recommend qualitative research to explore the underlying factors that contribute to the public’s concerns with international data sharing that would enhance global health.

**Supplementary Information:**

The online version contains supplementary material available at 10.1186/s12910-022-00855-z.

## Introduction

Biobanks collect, store, and distribute biospecimens and associated health data to investigators to conduct genomic research. Such research facilitates the identification of numerous genetic variants associated with chronic and widespread diseases such as cardiovascular diseases, diabetes, and Alzheimer’s disease [1]. Genomic research can also develop the field of precision or personalized medicine [2]. Furthermore, obtaining sufficient and representative samples for genome-wide association studies (GWAS) would be difficult without the development of biobanks [3]. Disproportionate contribution of biospecimens between low- and middle-income countries (LMICs) and high-income countries (HICs) to GWAS coupled with the lack of equitable partnerships and insufficient capacity building will lead to an equity gap in genomics health research [4].

However, biobanks have been limited to HICs and have infrequently been established in LMICs, primarily due to differences in infrastructure, training, and financial sustainability between HICs and LMICs [5] [6]. Additionally, a meaningful contribution of LMICs to the global database depends on the public awareness and acceptance of the importance of biobanks and consequently, their willingness to donate their biospecimens and associated health information to research. It is therefore essential to recognize factors that influence the commitment of the public to participate in biobank research [7]. Commentators emphasize that such factors might include knowledge of the types of future research performed on the donated samples, the extent of data sharing with national and international researchers, the scope of commercialization, the extent to which individual results are returned to participants, the right to withdraw biospecimens from future research, and the extent of data security, privacy, and trust [8] [9]. Several studies also indicate that demographic variables, such as gender, education level, and socioeconomic status might play a role in the willingness to donate [10–13]. The type of informed consent used in biobank research might additionally prove to be relevant in the decisions of individuals to donate their biospecimens [14–17]. Thompson and McNamee propose six possible models of informed consent that biobanks can implement, namely, verbal consent, blanket consent, broad consent, meta consent, dynamic consent, and waived consent [18].

Regarding public perspectives with participating in biobank research, studies performed in LMICs from the Arab Middle East region, specifically Egypt, Jordan, and Morocco, have reported disparate findings. These studies showed that the willingness of the public to participate in biobank research ranged between 43 and 85%, in these countries [19–24]. For example, Ahram et al. reported that although there was little knowledge of biobanks among Jordanians, approximately two-thirds indicated their willingness to donate samples and data for biobank research [20]. Factors associated with a willingness to participate in biobanking included younger age, higher education levels, the prospects of returning individual research results, and religious permission, whereas negative factors included future research that was left unspecified and the likelihood of being recontacted for informed consent. Finally, Lhousni and colleagues showed in a Moroccan study that 80.7% of participants expressed their willingness to participate in biobank research [23]. Willingness to participate in biobanks was significantly associated with gender and age, while the main barriers to participation in biobank research included the lack of trust in biomedical research and concerns about their privacy.

In contrast, Abou-Zeid and colleagues showed that among 600 participants from rural and urban centers in Egypt [19] less than half (43.5%) indicated they would donate their samples for future genetic research. In their study, respondents were more favorable toward having their blood samples exported to other Arab countries (62.0%) compared with countries in Europe (41.8%, p < 0.001). A large majority (89%) expressed the desire that they should be notified of results that are relevant to their health. In a more recent study performed in Egypt, 85.3% indicated they would donate samples to biobanks [22]. Most (91.1%) reported, however, that the researchers must maintain the privacy of their health information during the research. Additionally, less than one-third agreed to share their samples with either researchers abroad (32.4%) or with pharmaceutical companies (27.8%).

The above studies show that regarding participation in biobank research, the public harbor concerns with data security, privacy, and trust and most expressed a desire to receive individual research results that might indicate a risk of future illnesses. However, there were mixed results between these studies involving the extent of permissible data sharing and the preferred consent model. Divergent results between the different populations in LMICs in the Arab Middle East endorse the heterogeneity of the social fabric among them. In essence, classifying the LMICs as belonging to a specific region, i.e., “the Middle East,” ignores the unique social, cultural, and religious differences between the different countries. In reality, the phrase “Middle East” represents a recent geo-political construct in which countries were grouped together merely by their geography despite the social heterogeneity between them [25]. Variable findings of the studies might also be due to differences in recruitment methods and differently worded questionnaire items that resist consistent interpretation.

Using a previously validated questionnaire [26], we aimed to concurrently explore the perceptions, attitudes, and willingness of the public to participate in biobank research among four distinct LMICs in the “Middle East” region. Our objectives included (1) to explore the association between perceptions, attitudes of the public toward biobank research, attitudes toward privacy and trust, and their willingness to participate in biobank research and (2) to determine the factors associated with the perceptions, attitudes, and willingness of potential participants to participate in biobank research (i.e., donate biospecimens and their health-related data). Our additional objectives included (3) to determine differences between the  countries of the Middle East regarding the "willingness to participate in biobank research" and (4) to determine differences in constructs between the different countries. These objectives and the corresponding null hypotheses appear in Additional file 1 (Research objectives of the study and the corresponding null hypotheses).

## Methodology

### Study design

We performed a cross-sectional study conducted between September 2020 and January 2021.

### Study population

We recruited the general populations from Egypt, Jordan, Morocco, and Sudan.

### Questionnaire

We used a previously validated questionnaire, see Additional file 2 (Final Questionnaire used in the survey study). The information sheet of the questionnaire included a list of definitions of essential terms related to biobanks. These terms included: biobanks, DNA, genetic disease, privacy, scientific research, research ethics committee, clinical trials, consent, and protection of informational privacy. For example, we defined biobanks as follows:

Biobanks: facilities where different biological samples such as bodily fluids (blood, urine, etc.) or tissue samples are stored. These biological samples are collected from healthy donors or patients along with information related to them, their health conditions, their donated samples, or, sometimes, their families. Researchers use these samples to find new diagnostic methods or treatments for various diseases, especially those whose treatment is currently difficult such as cancer.

For the other definitions included in the information sheet, see Additional file 3 (Information Sheet and Informed Consent Form Presented to the Participants.

The questionnaire incorporated a sociodemographic part and additional sections representing several constructs. We define a construct as “an underlying theme, or subject matter that one intends to measure by using survey questions”. We used the following constructs: (a) perceptions about biobanks; (b) aspects of biobank research that affect willingness to donate; (c) attitudes toward biobank research; (d) attitudes toward trust and privacy, and (e) willingness to participate in biobank research (i.e., donate specimens). We used a Likert scale to categorize response items for each of the constructs. The Likert scale is commonly used in social science survey research to measure entities that cannot be expressed numerically, such as attitudes and opinions [27].

Response items regarding perceptions consisted of a 3-point scale (yes, no, not sure). Responses to the construct “aspects of biobank research that affect willingness to donate” consisted of a 5-point Likert scale: “very important”, “important”, “moderately important”, “somewhat important”, and “not important”. Responses of the constructs regarding “attitudes toward biobank research” and “attitudes toward trust and privacy” consisted of a 6-point Likert scale (strongly agree, agree, no opinion, disagree, strongly disagree, and I do not understand). Responses to the construct “willingness to participate in biobank research” consisted of a 5-point Likert scale (definitely yes, probably yes, not sure, probably not, and definitely not”).

We defined “perception” as “the subjective process of acquiring, interpreting, and organizing sensory information” and distinguish it from an “attitude”, which represents the evaluation of one’s perception or one’s subjective feeling or emotion toward a fact or state. Basically,  “perception comes first, and then the attitude, or behavior based on the perception, comes later” [28].

The questionnaire also contained different scenarios for participation in a biobank based on the type of informed consent form. Five scenarios were presented to the respondents: broad consent with coded database, broad consent with irreversibly anonymized database, tiered consent with coded samples, tiered consent with irreversibly anonymized database, and recontact for future research consent. The scenarios included definitions of the different consent forms as shown in the copy of the questionnaire that is included in Additional file 2 (Final Questionnaire used in the survey study). For each scenario, respondents were asked to choose from a 5-point Likert scale that included responses ranging from “definitely yes” to “definitely no” and a “not sure” response. Respondents were asked to provide their responses to each scenario and hence, the types of consent chosen was not mutually exclusive.

### Recruitment methods

In Egypt, Morocco, and Jordan, we distributed the questionnaires through different social media platforms, e.g., Facebook, LinkedIn, and WhatsApp, all of which are commonly used in the Middle East. Social media advertisements were also purchased to recruit participants in Egypt, reaching over 100,000 individuals. In Sudan, data collectors distributed the questionnaire in paper to potential participants in the markets and from health facilities. Data collectors did not conduct any face-to-face interviews, although they read the questions to individuals who had illiteracy.

After the participants received information about the survey, they proceeded to complete the questionnaire. The target audience was adults 18 years and older of both genders and of all educational backgrounds and economic statuses. Participants were informed that their participation was voluntary, that they could withdraw at any time, and that they could omit any question.

### Sample size

The sample size was calculated based on the percentage of potential participants who expressed willingness to participate in biobanking reported from a previous population-based study conducted in Jordan [20]. The sample size was determined using Epi info, version 3.5.1, 2008. Based on the confidence level of 95%, a power of 80%, and the 64% of participants who would show a willingness to participate in biobanking, a design effect of 2, and 20% missing responses the required minimum sample size was calculated to be 850 participants.

### Statistical Analysis

We calculated a total score for each construct of the questionnaire. For the “perceptions” construct, we assigned a score of 1 point to responses that reflected accurate perceptions and 0 points were given for inaccurate perceptions and the “not sure” responses. The authors determined which items reflected an accurate perception based on their knowledge of the current literature. The total score was calculated by the simple addition of the responses from the study population. The potential range of the scores was 0-to 12 with higher scores reflecting more accurate perceptions.

For the other constructs, we collapsed the two response items at either end of the Likert scale into one category. For example, for the “aspects” construct, we collapsed the response items of “very important” and “important” into one category (important) and “slightly important” and “not important” into another category (not important). For both “attitudes” constructs, we collapsed the response items of “strongly agree” and “agree” into an “agree” category and the response items of “disagree” and “strongly disagree” were collapsed in a “disagree” category. Negatively worded statements that did not reflect an attitude consistent with normative beliefs/practices were reversed coded.

We assigned a score of 3, 2, and 1 to each of the three categories of these three constructs. Higher scores were attached to the “important” and “agree” categories. The total score for each construct was calculated by the simple addition of the responses from each respondent. The potential range of the scores for the “aspects” construct was 10–30, with higher scores reflecting “aspects” that are more important in determining whether to participate in biobank research. The potential range of the scores for the “attitudes toward biobank research” construct was 11–33 with higher scores representing attitudes that were more reflective of normative beliefs/practices. The potential range of the scores for the “attitudes toward trust and privacy” construct was 3–15 with higher scores consistent with attitudes that reflect normative attitudes. For the “willingness construct” the two response categories at either end of the scale were collapsed into one category each (“definitely/probably yes” and definitely/probably not”, respectively). Scores ranged from 1 to 3 points with the higher point value attached to “definitely/probably yes” category. The potential range of the total score of the participants was from 5 to 15 with higher scores reflecting a greater willingness to participate in biobank research. Data were summarized as frequencies and percentages and mean ± standard deviation (SD) for the aggregate scores.

We used the Turkey test to perform post-hoc multiple comparisons. We constructed a multiple linear regression model to identify the independent predictors of the construct: “willingness to participate in biobank research.” All variables with p values < 0.10 in the bivariate analysis were included in the model. The Statistical Package for the Social Sciences (SPSS), version 20.0, for Windows was used. The tests were two-tailed, and p values ≤ 0.05 were considered to indicate statistical significance.

## Results

### Demographics of the study population

We recruited 967 participants; the average age was 33 ± 11 years, and the range was 18–73. The majority (61.3%) were from Egypt and males constituted 56.3% of the total sample. Of our participants, 48.2 had never been married and most (83.6%) did not have children. As for health status, 86.6% of respondents described themselves as healthy and only 2.3% had a history of cancer; 83% lived in urban areas. The majority indicated they were Muslims (95.8%), and most thought they were somewhat religious (70.4%), while approximately 15% considered themselves to be “very religious”. More than 70% were at the graduate or post-graduate level. Regarding awareness of the term “biobank”, more than 70% said either “no” or “not sure”. Regarding prior research participation, 16.2% had participated in research and 35.5% said they had donated a sample for research purposes. (See Table 1 for further details).

**Table 1:** Demographic characteristics of participants (n = 967)

Demographic variable	n	%
*Country*		
Egypt	593	61.3
Morocco	68	7.0
Jordan	123	12.7
Sudan	183	18.9
*Gender*		
Female	423	43.7
Male	544	56.3
*Marital status*		
Widowed	12	1.2
Divorced	27	2.8
Never married	466	48.2
Married	462	47.8
*Children*		
No	808	83.6
Yes	159	16.4
*Medical condition*		
Healthy	837	86.6
Disease other than Cancer	108	11.2
Cancer	22	2.3
*Residence*		
Rural	164	17.0
Urban	803	83.0
*Degree of religiosity*		
Not religious at all	31	3.2
Not very religious	117	12.1
somewhat religious	681	70.4
Very religious	138	14.3
*Education*		
No formal education	4	0.4
Less than primary	11	1.1
Middle school	39	4.0
Technical education	77	8.0
High school	152	15.7
Graduate	546	56.5
Post-graduate	138	14.3
*Awareness of the term biobank*		
Not sure	169	17.5
No	509	52.6
Yes	289	29.9
*Previous participation in research studies*		
No	872	83.8
Yes	169	16.2
Types of research studies of previous participation*		
Clinical trials	23	13.6
Sample donation	60	35.5
Gene study	32	18.9
Questionnaire/Interview	148	87.6
Do not know	20	11.8
*Age*		
Mean ± SD (Range)	33 ± 11 (18–73)	

*Responses are not mutually exclusive

### Perceptions about biobanks

The total mean total perception score was 4.38 ± 1.68 (maximum possible score is 12) indicating a less than moderate accurate perception of biobanking practices. This score results from many participants answering “not sure” for many items. For example, regarding specific perceptions toward biobanks, almost three-quarters were “not sure” whether biobank research would lead to better medical treatments in the future or improve an individual's health (78.9 and 72.3%, respectively). Regarding data sharing, 66.2% were “not sure” that biological specimens can be shared with local researchers.

Finally, a sizable minority of the participants held inaccurate perceptions of biobanks. For example, 32.2% thought that biobank research would only benefit private drug companies and 32.4% indicated that people will spend monies to donate biological samples. Another inaccurate perception included the possibility that a person might be cloned from donated biospecimens, which was believed by 44.7% of respondents. Approximately a quarter of the respondents (26.8%) believed that biological samples could be used to produce biological weapons. See Table 2 for further details.

**Table 2:** Participants’ perceptions about biobanks (n = 967)

	Yes (n)	%	No (n)	%	Not sure (n)	%
*Donation*						
Biobank research can lead to better medical treatments for future generations	32	3.3	172	17.8	763	78.9
Biobank research can lead to improvement in an individual’s health	68	7.0	200	20.7	699	72.3
People will have to spend monies to donate biological samples*	313	32.4	361	37.3	293	30.3
Biobank research will only benefit private drug companies*	321	32.2	464	48.0	182	18.8
*Storage*						
People who donate their biological samples will not be able to request to have their samples destroyed in the future*	262	27.1	285	29.5	420	43.4
Biological specimens given to a biobank can be sold to anyone*	387	40.0	233	24.1	347	35.9
*Privacy*						
Personal medical information stored in a biobank might be revealed to unauthorized people*	443	45.8	204	21.1	320	33.1
*Data Sharing*						
Biological samples can be shared with researchers in other institutions in my country	95	9.8	232	24.0	640	66.2
*Research*						
Researchers are more interested in making money from donated biological samples than doing good research*	412	42.6	210	21.7	345	35.7
A person might be cloned if he/she donates a biological sample to a biobank*	432	44.7	158	16.3	377	39.0
Biological samples will be used for the production of biological weapons*	259	26.8	318	32.9	390	40.3
*Return of results*						
Researchers will contact people if the analysis of their biological specimens shows risk for disease	63	6.5	247	25.5	657	67.9
Mean total perception score (mean ± SD)	4.38 ± 1.68					

*Reversed scored

### Aspects of biobank research that affect willingness to donate

The mean total score for this construct was 12.7 ± 3.2 (maximum possible score is 30), which reflects that most respondents “indicated” that many of the “aspects” items presented to them were “not important” or only “slightly important” in a decision to donate. These included “Future research on my biological samples could improve healthcare for people in the future” (82.4%); “future research… will be reviewed by an ethics committee” (88.5%), “my religion approves of my donating biological samples” (85.5%), and “I will be able to obtain the genetic results from the analysis of my biological samples” (84.9%). Finally, more than 80% felt that the possibility that “Future research on my biological samples could improve healthcare for people in the future” was not important. In contrast, approximately 50% thought that data sharing with international researchers was an important aspect (combination of “important” and “moderately important”). See Table 3 for further details.

**Table 3:** Aspects of biobank research that affect willingness to donate. (n = 967)

	Important	Moderately Important	Not important
*Donation*			
Future research on my biological samples could improve healthcare for people in the future	50 (5.2)	120 (12.4)	797 (82.4)
Future research on my biological samples will be reviewed by an ethics committee	41 (4.2)	70 (7.2)	856 (88.5)
My personal health will improve from my donation	129 (13.3)	180 (18.6)	658 (68)
My religion approves of my donating biological samples	77(8)	63 (6.5)	827(85.5)
*Privacy*			
My medical information will remain private	35(3.6)	41 (4.2)	891(92.1)
if the analysis of my biological samples reveals any stigmatizing information about me, this will be kept private	41 (4.2)	52 (5.4)	874 (90.4)
*Data Sharing*			
My biological samples and medical information will be shared with researchers who are from other countries	267(27.6)	216 (22.3)	484(50.1)
Access to my biological samples and medical information in the biobank will be strictly controlled by an oversight committee	51(5.3)	71 (7.3)	845 (87.4)
Researchers outside of my institution will not receive any biological samples or medical information that directly identifies me	90 (9.3)	95 (9.8)	782(80.9)
*Return of results*			
I will be able to obtain the genetic results from the analysis of my biological samples	69 (7.1)	77 (8.0)	821 (84.9)
Mean total “aspects” score, mean ± SD	12.7 ± 3.2		

### Attitudes toward biobank research

The mean score for “attitudes toward biobank research” was 15.07 ± 3.64 (maximum possible score is 33) reflecting a less than moderate score for holding attitudes toward biobank research that reflect normative beliefs/practices. For example, less than 5% of respondents agreed that people should donate samples to improve the health of future generations. Furthermore, less than a quarter (23%) agreed that donated samples could be shared with researchers at institutions in other countries; less than 5% agreed that researchers must maintain the privacy of a donor’s medical information and only 23.1% approved that legal authorities should have the right to access personal data, when necessary. Finally, less than 10% of the respondents agreed that research results should be returned to donors even if it reveals a treatable (2.3%) or untreatable disease (6.1%). See Table 4 for further details.

**Table 4:** Participants’ attitudes toward biobank research (n = 967)

	Agree	Neutral	Disagree
*Donation*			
People should donate biological samples to improve the health of future generations	28 (2.9)	144 (14.9)	795 (82.2)
People should donate biological samples even if there will not be a direct health benefit to them	166(17.2)	206 (21.3)	595(61.5)
People who donate biological specimens should receive financial compensation that is in addition to any travel expenses	171(17.7)	223 (23.1)	573(59.3)
*Storage*			
If people change their minds, they should have the right to withdraw their consent for the use of their biological samples	92(9.5)	119 (12.3)	756(78.2)
*Privacy*			
Researchers must maintain the privacy of a donor’s medical information when they perform research	17(1.8)	82 (8.5)	868(89.8)
*Data sharing*			
It is acceptable for biological samples to be shared with researchers at other institutions in my country	112(11.6)	153 (15.8)	702(72.6)
It is acceptable for samples to be shared with researchers at institutions in other countries	222(23)	195 (20.2)	550(56.9)
The legal authorities should have the right to obtain my genetic results when necessary	223(23.1)	174 (18.0)	570(58.9)
Researchers should receive governmental approval prior to exporting samples out of the country	72(7.4)	122 (12.6)	773(79.9)
*Return of results*			
If the analysis of my biological specimens reveals a disease that can be treated or prevented, then either I or my doctor should be informed of these results	22(2.3)	76 (7.9)	869(89.9)
Even if the analysis of my biological specimens reveals a genetic disease that cannot be treated or prevented, I still want to be informed of these results	59(6.1)	91 (9.4)	817(84.5)
Mean total “attitudes toward biobank research” score, mean ± SD	15.07 ± 3.64		

### Attitudes toward “trust and privacy”

The mean score for this construct was 5.34 ± 1.59 (maximum possible score is 15) reflecting a low score for having trust in biobank managers and believing that privacy would be protected by medical physicians and researchers. For example, more than half of the respondents indicated a privacy concern with sharing their medical information with their physicians or researchers (50.2% and 56.7%, respectively). Regarding “trust”, less than 20% strongly agreed or agreed that they would trust individuals in charge of biobanks. See Table 5 for further details.

**Table 5:** Participants’ attitudes toward trust and privacy (n = 967)

	Agree	Neutral	Disagree
I worry about the privacy of my medical information when I share it with my doctor	485(50.2)	141 (14.6)	341(35.3)
I worry about the privacy of my medical information when I share it with researchers	548(56.7)	171 (17.7)	248(25.6)
I trust the individuals in charge of biobanks	184(19.0)	392 (40.5)	391(40.4)
Mean total “attitudes toward trust/privacy” score mean ± SD	5.34 ± 1.59		

### Willingness to participate in biobank research

The mean “willingness” score was 7.1 ± 3.0 (maximum possible score is 15) reflecting a less than moderate degree of “willingness” to participate in research. Almost three-quarters of respondents indicated that they would refuse to give any type of sample to participate in research and 80.2% would refuse to provide medical information. Only 19.6% of the participants indicated that they would participate in genetic research. See Table 6 for further details.

**Table 6:** Participants’ “willingness to participate in biobank research” (n = 967)

	Definitely yes	Not sure	Definitely not
If you are asked to give your medical information for research, would you agree to do it?	88 (9.1)	103 (10.7)	776 (80.2)
If you are asked to give saliva for research, would you agree to do it?	130 (13.4)	100 (10.3)	737 (76.2)
If you are asked to give a urine sample for research, would you agree to do it?	143 (14.8)	98 (10.1)	726 (75.1)
If you are asked to give a blood sample for research, would you agree to do it?	154(15.9)	113 (11.7)	700 (72.4)
If you are asked to participate in genetic research, would you agree to do it?	189 (19.5)	188 (19.4)	590 (61.0)
Total “willingness” score, mean ± SD	7.1 ± 3.0		

### Preferences for types of informed consent

**Fig. 1:**
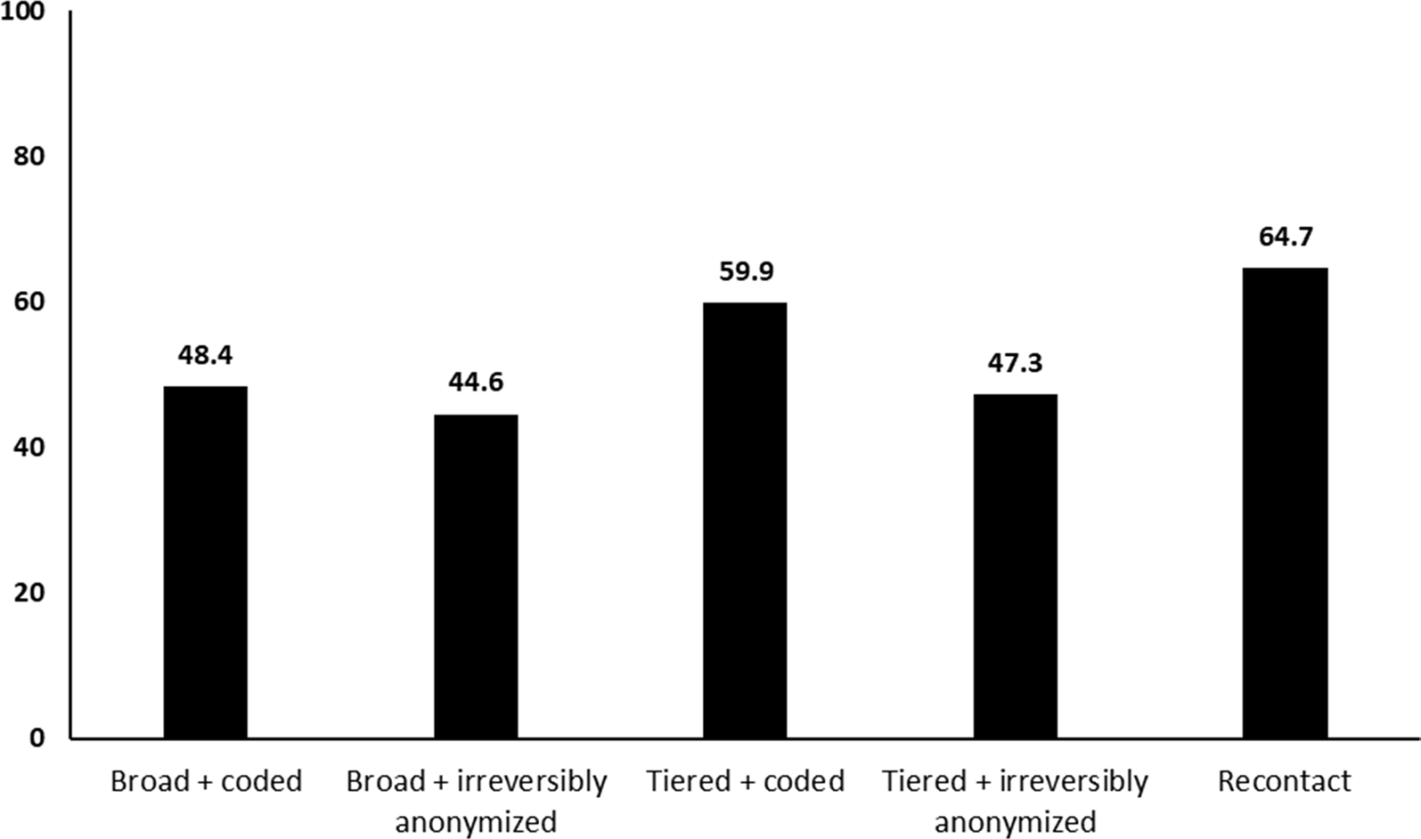
Participants’ preferences towards types of informed consent (agree + strongly agree). (n = 967)

Regarding the preference for the type of informed consent when donating a biospecimen, approximately 60% favored two types of informed consent. One included the use of tiered consent with coded samples (59.9%), and the other was recontacting donors for future research (64.7%). Less than a majority favored a consent model that is either broad and coded, broad and irreversibly anonymized, or tiered and irreversibly anonymized (see Fig. 1).

### Analysis between the different constructs

Correlation analysis showed a significant relationship between the construct “willingness to participate in biobank research” and all the other constructs: “perceptions”, “attitudes toward biobanks” and “attitudes toward privacy and trust”. All correlations were significant; positive and negative sign shows the dimension of relationship. There was a negative weak relationship between “willingness to participate” and “perceptions” (r = − 0.111). There was a positive moderate relationship between “willingness to participate” and “attitudes toward biobank research (r = 0.466). There was a positive weak relationship between “willingness to participate” and “attitudes towards trust and privacy” (r = 0.066). Further details are shown in Table 7. An additional correlation analysis between “perceptions” and “attitudes toward biobank research” showed a significant weak indirect relationship (r = − 0.076, p = 0.018).

**Table 7:** Correlation between the different constructs (Pearson correlation)

	Perceptions about sample donation in biobanks	Attitudes towards participation in biobanking research	Attitudes toward privacy and trust
Willingness to participate in biobank research	− 0.111** p = 0.001 n = 967	0.466** p = 0.0001 n = 967	0.066* p = 0.041 n = 967

**Correlation is significant at 0.01 level; *correlation is significant at 0.05 level

  As it is evident from the aforementioned results that there are statistically significant relationships between the constructs, we reject our first null hypothesis: **H1**: There is no association between “willingness to participate in biobank research” and “perceptions about biobanks”, “attitudes toward biobank research”, and “attitudes toward privacy and trust.”

Significant associations were shown between several of the independent variables and the different constructs (see Table 8). For example, as a group, the countries showed significant associations with all of the constructs and there were significant differences between the countries for each specific construct. For instance, for the "perception" construct, Jordan and Sudan had higher scores compared with Egypt and Morocco. The scores between Sudan and Egypt and between Sudan and Morocco were statistically significant by the Turkey test for multiple comparisons (p < 0.0001 and p < 0.045; respectively). Egypt had higher scores on “attitudes toward biobank research” compared with the other three countries. The score between Egypt and Jordan was statistically significant by the Turkey test for multiple comparisons (p < 0.003). Morocco had statistically higher scores on “attitudes toward trust and privacy” compared with the other three countries (p < 0.0001, p < 0.003, and p < 0.0001for Egypt, Jordan, and Sudan; respectively). Egypt had higher scores compared with the other countries for the construct "willingness to participate in biobank research", but the differences were not significant on the multiple comparisons test (See Additional file 4: Post Hoc multiple comparisons test).

**Table 8:** Association between participants’ demographics and questionnaire domain scores (n = 967)

	Perceptions about Biobanks		Aspects of biobank research that affect willingness to donate biospecimens		Attitudes toward biobank research		Attitudes towards trust and privacy		Willingness to participate in biobank research	
	Mean ± SD	p value	Mean ± SD	p-value	Mean ± SD	p value	Mean ± SD	p value	Mean ± SD	p value
*Country*										
Egypt	4.2 ± 1.56	0.000	12.9 ± 3.7	0.000	15.4 ± 4.0	0.001	5.19 ± 1.56	0.000	7.25 ± 3.1	0.020
Morocco	4.2 ± 1.56		11.9 ± 1.7		14.4 ± 3.2		6.24 ± 1.77		6.4 ± 2.7	
Jordan	4.56 ± 1.8		11.8 ± 1.7		14.19 ± 2.6		5.4 ± 1.47		6.5 ± 2.7	
Sudan	4.8 ± 1.6		13.0 ± 2.6		14.74 ± 2.7		5.34 ± 1.62		7.05 ± 3.0	
*Area*										
Rural	4.476 ± 1.68	0.43	13.06 ± 3.34	0.22	14.98 ± 3.75	0.69	5.67 ± 1.67	0.001	6.84 ± 2.83	0.27
Urban	4.356 ± 1.68		12.71 ± 3.23		15.10 ± 3.60		5.25 ± 1.58		7.125 ± 3.07	
*Gender*										
Female	4.396 ± 1.63	0.80	12.44 ± 2.94	0.001	14.95 ± 3.43	0.32	5.433 ± 1.65	0.06	7.09 ± 3.05	0.91
Male	4.369 ± 1.73		13.03 ± 3.46		15.18 ± 3.78		5.241 ± 1.57		7.07 ± 3.02	
*Children*										
No	4.437 ± 1.68	0.25	12.688 ± 2.75	0.35	15.158 ± 3.60	0.51	5.333 ± 1.62	0.89	6.977 ± 2.91	0.25
yes	4.314 ± 1.69		12.882 ± 3.76		15.004 ± 3.67		5.319 ± 1.58		7.199 ± 3.17	
*Previous participation in research*										
No	4.389 ± 1.69	0.73	12.86 ± 3.35	0.06	15.123 ± 3.70	0.49	5.349 ± 1.61	0.33	7.208 ± 3.09	0.001
Yes	4.3392 ± 1.65		12.34 ± 2.69		14.906 ± 3.29		5.2137 ± 1.56		6.4215 ± 2.59	
*Age group*										
< 40	4.388 ± 1.65	0.81	12.90 ± 3.47	0.02	15.225 ± 3.79	0.02	5.337 ± 1.61	0.69	7.127 ± 3.01	0.36
> 40	4.357 ± 1.80		12.33 ± 2.34		14.614 ± 3.00		5.289 ± 1.58		6.913 ± 3.10	
*Medical condition*										
Healthy	4.3944 ± 1.70	0.55	12.834 ± 3.29	0.16	15.125 ± 3.66	0.41	5.338 ± 1.62	0.57	7.085 ± 3.03	0.87
Non-Healthy	4.2996 ± 1.62		12.408 ± 3.02		14.8467 ± 3.43		5.253 ± 1.46		7.038 ± 3.06	
*Education level*										
Less than University	4.47 ± 1.72	0.29	13.074 ± 3.48	0.06	15.44 ± 3.91	0.04	5.533 ± 1.67	0.01	7.551 ± 3.45	0.001
University and higher	4.345 ± 1.67		12.654 ± 3.15		14.93 ± 3.50		4.0745 ± 9.95		6.883 ± 2.82	
*Marital status*										
ever married	4.3572 ± 1.71	0.64	12.798 ± 3.58	0.83	14.994 ± 3.69	0.40	5.285 ± 1.57	0.40	7.11 ± 3.1	0.74
Never married	4.4076 ± 1.66		12.753 ± 2.87		15.1888 ± 3.57		5.371 ± 1.63		7.045 ± 2.95	
*Degree of religiosity*										
Not and Less than very religious	4.344 ± 1.75	0.77	13.358 ± 3.28	0.01	15.054 ± 3.62	0.90	5.3175 ± 1.57	0.94	7.047 ± 3.07	0.89
Very religious	4.388 ± 1.67		12.672 ± 3.24		15.094 ± 3.64		5.328 ± 1.61		7.084 ± 3.03	
*Awareness of the term “biobank”*										
No	4.352 ± 1.69	0.41	12.99 ± 3.45	0.00	15.248 ± 3.73	0.03	5.325 ± 1.61	0.98	7.305 ± 3.14	0.00
Yes	4.449 ± 1.68		12.27 ± 2.68		14.712 ± 3.36		5.328 ± 1.60		6.546 ± 2.70	

None of the demographic variables showed significant associations with the construct “perceptions about biobanks.” Being “age < 40” and having at an “education level less than university” had significant higher scores with “attitudes toward biobank research”, (p < 0.02 and p < 0.04; respectively); whereas stating “not being aware of biobanks” had a significantly higher score compared with being “aware”, p < 0.001). Regarding “attitudes regarding trust and privacy”, significantly higher scores were revealed for living in a “rural area” compared with “urban living” (p < 0.001); and with having a degree below the level of a university degree compared with a degree at or higher than the university level (p < 0.01). Significantly higher scores with “willingness to participate in biobank research” were associated with “not having previous participation in research” and “having a less-than-university degree”. See Table 8 for further details.

Associations between participants’ demographics and the percentage of “strongly agree” and “agree” with the individual questionnaire items are shown in Additional file 5 (Bivariate analysis between demographic variables and individual question items of the construct "attitudes toward biobank research)  and Additional file 6 (Bivariate analysis between demographic variables and individual question items of the constructs). 

While the aforementioned results showed several statistically significant associations between the demographics and the different constructs, correlations analysis demonstrated that these associations had a weak relationship (all r < 0.2). (See Additional file 7: Correlation analysis between demographics and constructs).

We, therefore, accept our second null hypothesis: **H2:** Demographics do not have a significant **impact** on perceptions, attitudes, and willingness to participate in biobank research.

Our above results showed that the differences between the “willingness to participate in biobank research"  between the four participating countries were not significant on the multiple comparisons test. Accordingly, we accept our third null hypothesis: **H3**: There are no significant differences in the "willingness of the public to participate in biobank research" between the four different countries.

However, we reject our fourth null hypothesis as there are variations in the other constructs between the four countries on the multople comparisons test: **H4**: The different countries do not have variations in the constructs (i.e., perceptions and “attitudes”) that accounts for the differences in willingness to participate in biobank research.

Correlation analysis between “willingness to participate in biobank research” and the other three different constructs of the four countries showed a moderate significant positive relationship between “attitudes toward biobank research” and “willingness to participate in biobank research” (all r > 0.42). The relationships between the “perceptions” and the “attitudes toward trust and privacy” constructs for all four countries demonstrated weak significant negative relationships. (See Additional file 8: Correlation between the different constructs of each country and the willingness to participate in biobank research).

### Predictors of willingness to participate in biobank research

There were several predictors for “willingness to participate in biobank research”. Residing in Egypt had a positive influence on “willingness to participate in biobank research”, while Jordan had a negative influence. Other predictors that had a negative influence on participation and included: “previous participation in research”, “having an education level at university or higher”, and “having an accurate perception of biobanks.” The construct “attitudes toward biobank research” had a positive influence on “willingness to participate in biobank research”. See Table 9 for further details.

**Table 9:** Multivariate linear regression analysis between independent variables against willingness to participate in biobank research (n = 967)

Independent variable	standardized coefficient (β)	95% CI	t	P value
*Country*				
Egypt	0.069	0.000–0.034	2.012	0.04
Morocco	− 0.037	− 0.246–0.072	− 1.071	0.284
Jordan	− 0.072	− 0.248–(− 0.015)	− 2.210	0.02
Sudan	− 0.016	− 0.125–0.074	− 0.498	0.61
Age (1 year)	− 0.12	− 0.109–0.074	− 0.374	0.70
Participants with children (Yes vs. No)	0.081	− 0.032–0.228	1.484	0.13
Previous participation in research (Yes vs. No)	− 0.086	− 0.201–(− 0.016)	− 2.302	0.02
Education (university and higher vs less than university)	− 0.065	− 0.162–(− 0.012)	− 2.2186	0.02
Marital status (Ever married vs. Never married)	0.046	− 0.073–0.185	0.849	0.39
Awareness of the term “biobank” (yes vs. no/not sure)	− 0.052	− 0.144–0.007	− 1.792	0.07
Perception of Biobanks (1 unit increase)	− 0.077	− 0.569–(− 0.092)	− 2.723	0.007
Attitudes towards biobanking research (1 unit increase)	0.399	0.612–0.851	12.048	0.001
Aspects of biobank research that affect willingness to donate (1 unit increase)	0.102	0.068–0.311	3.059	0.002
Attitudes about trust and privacy (1 unit increase)	− 0.006	− 0.070–0.056	− 0.216	0.82

## Discussion

Our study, involving several LMICs in the Arab region of the Middle East, demonstrated that only a minority of the public (less than 30%) were aware of the term “biobanks” and only a small proportion of our participants (less than 20%) were willing to participate in biobank research.

Our participants’ level of awareness regarding biobank research compares with other studies performed in the Middle East. For example, in a study performed in Jordan, only approximately 26% of a representative sample of the population knew what the term “biospecimen” represented [20]. These results are confirmed by recent studies from Egypt [22] Morocco [23], and Jordan [24], in which the rates of awareness were 53.7%, 32.4%, and 28.5%; respectively.

The low awareness of the public regarding biobanks that we observed was accompanied by a low percentage of the public’s willingness to participate in biobank research. This result contrasts with recent studies from Egypt, Jordan, and Morocco, which revealed a higher willingness of the public to participate in biobank research that ranged between 65 and 85% [22–24]. This finding of low awareness coupled with high willingness from the other studies compares with other studies investigating the views of the public toward biobanks. For example, Mezinska and colleagues surveyed the Latvia public and showed that despite only a quarter of the participants being aware of biobanks, almost a half were willing to donate blood samples to a biobank [29].

We are left with the task to explain the divergent results between our study showing low awareness and low willingness with the other studies showing low awareness yet a high willingness to donate biospecimens. One explanation might ensue from the difference in methodologies used to recruit and obtain survey responses in the different studies. Specifically, our study was conducted mainly online, whereas the other studies we mentioned above used face-to-face interviews, which might have led to an “interviewer effect” leading to a social desirability bias in face-to-face methodologies [30, 31]. By way of explanation, face-to-face interviews could cause desirability or acquiescence bias since participants would give an answer that was to satisfy the interviewer. This bias would not occur with surveys conducted online suggesting that online responses might reflect better the true intentions of the participants. It is worth mentioning that although the questionnaires were distributed in-person in Sudan, face-to-face interviews were avoided.

The one study from Jordan that also used an online methodology stands in contrasts to the study herein insofar that despite a low awareness, the Jordan investigators showed a high willingness of the public to donate a biospecimen (more than 85%). Two factors might explain the differences between the results between the Jordan study and ours. First, almost 50% of the participants in the Jordan study revealed prior participation in research, which contrasts with only 16% in our study. More importantly, greater than 75% of the participants from Jordan agreed that they trust research and researchers, which contrasts with the less than 20% of our participants who revealed trust in biobank managers. Furthermore, we showed that most of our participants harbored concerns about doctors or researchers with protecting the privacy of their information.

The significance of trust regarding willingness to donate biospecimens and associated health data corroborates the importance of trust regarding biobank research shown in other studies. For example, Pawlikowski and colleagues explored the associations between the willingness to donate samples to biobanks and selected psychological variables. Of the variables studied, one included trust in doctors and scientists. [32]. Similarly, Iott and colleagues showed that patients’ trust in physicians is associated with their information-sharing concerns or behaviors [33]. Dive and colleagues reported the results of the Australian public attitudes toward the networking and globalization of biobanks [34]. Using quantitative and qualitative methods, they explored factors that may contribute to or threaten trust. Their results indicated a generally high level of trust in biobanks and medical research more broadly, but key factors that can reduce the perceived trustworthiness of biobanks involved issues related to commercialization and participation in global networking. They recommended that robust ethical oversight and governance standards can promote trust in global biobanking.

Importantly, our results parallel those of Gaskell et al. who showed that countries such as Austria and Greece were characterized by the lowest levels of willingness to participate and the lowest level of trust in their government [35, 36]. In focus groups, “Greek participants explained that they don’t trust their country’s political system and therefore worry that the data would fall into the wrong hands” [35]. Individuals from the focus groups expressed the most concern about privacy and about what their genetic data might be used for. Greece also had the highest number of people selecting ‘narrow consent’.

Other studies have found that trust towards researchers depended on the nature of the entity where they were employed. For example, concerns towards researchers working for commercial and private (e.g., for-profit) have been demonstrated in many studies [12, 34, 37, 38]. However, the reluctance with data sharing with academic researchers has been variable. A high level of mistrust regarding academic researchers was shown in one study from the US [39]. In contrast, a high level of trust was reported in an Australian study [40] and in a study conducted in the US [41].

Trust and privacy are intimately related as one could infer that trust plays a role in the willingness of individuals to share their private information. Previously, Abdelhamid and colleagues demonstrated that privacy concerns had the most influence on individuals' intentions to share their “protected health information” electronically with health care providers [42]. Similarly, most of our respondents indicated that they could not trust their doctors or researchers to protect the privacy of their information. Finally, Makhlour and colleagues showed in their study from Jordan that concerns with one’s privacy and confidentiality had weak, but statistically significant negative correlation with a willingness to donate samples for biobank research.[23].

The importance of trust and privacy protection probably played a role in our respondents’ choices for informed consent. A majority selected either the option of re-consenting for every new secondary biospecimen research (study-specific consent) or a tiered consent model with coded samples (categorical consent). Both types of consent offer the most degree of autonomy and a high level of control compared with broad consent. Such choices reflecting the desire for more control over choices infer a lack of trustworthiness with the other options that offer less control.

Studies conducted in the Arab region of the Middle East (e.g., Jordan and Morocco) showed a preference for broad consent [23, 43, 44]. However, in a questionnaire study involving 600 Egyptians, many participants favored a tiered consent model or a preference for recontact [19]. Other studies exploring the preferred type of consent have shown a lack of consensus [41, 45], while others have shown a preference for broad consent [34, 46]. In a US study involving an exploratory mixed methods design, Simon and colleagues showed that broad consent (i.e., research-unspecific consent) was preferred over categorical and study-specific consent models for purposes of approving future research use [53]. An overview of the studies that assessed the public preference for consent forms for biobanks suggests no consensus even within the same population [47].

To further explain our results regarding the low rate of willingness, we investigated the relationship between several of the constructs and willingness to donate biospecimens. We showed that “perceptions” about biobanks had a negative association with a willingness to participate in research. Specifically, individuals who had more accurate perceptions of biobanks were less willing to participate in biobank research. These findings regarding the impact of the degree of accurate perceptions on “willingness” could be analogous to results regarding educational levels. Specifically, our study showed that a lower educational level was a predictor of willingness to participate in biobanks. Additionally, we showed that a lower educational level was associated with “attitudes toward biobank research” and “attitudes toward trust and privacy”. In contrast, while individuals living in rural areas had higher scores on “attitudes toward trust and privacy” compared with individuals in urban areas, individuals from rural areas did not have higher scores on “attitudes toward biobank research” and a “willingness to donate” compared with their urban counterparts. Our results with “lower educational levels” find support in other studies. For example, Labib et al. [48] and Nilstun and Hermerén [49] showed that individuals with "higher levels of education have restrictive attitudes towards donating samples to biobanks. The downstream negative effects of education on biobank participation is similar to the observation that increasing knowledge of genetic testing does not necessarily increase the enthusiasm for genetic testing, rather there is expressed skepticism [50, 51]. In contrast, Mezinska and colleagues reported in Latvia that higher education levels were associated with a higher degree of sample donation.

Although we showed that the construct “perceptions” was negatively correlated with “attitudes toward biobank research”, our study is noteworthy as correlation analysis revealed that “attitudes toward biobank research” had a significant positive correlation with a “willingness to donate”. Additionally, for each of the countries in our study, “attitudes toward biobank research” also had a significant positive correlation with a “willingness to donate”. The significance of attitudes and willingness to donate relies on the work of social scientists who explored the relationship between attitudes and behavior. Specifically, Ajzen and Fishbein used two components to predict intentions to explain behavior. One component measures the person's perceptions of what other people expect him or her to do (i.e., social norms) and the other component involved a motivation to comply with these expectations [52].

We also investigated “aspects” that affect willingness to donate biospecimens in our study. Of the “aspects” that affect willingness to donate biospecimens that we explored in this study, we found that sharing biospecimens and data with researchers from other countries was “important” with almost 50% of our respondents. This result is consistent with our finding that approximately 60% of our participants “disagreed” with the attitude that it is acceptable for samples to be shared with researchers at other institutions. The essence of biobanks is sharing of biospecimens and related information with other researchers. The alleged concern we discovered with data sharing in the international context corroborates the finding of Abou-Zeid and colleagues who showed in a survey conducted in 2010 that Egyptians’ reluctance to share biospecimens and data with Western countries was higher than with researchers in Arab countries [19]. Similarly, in an international study, Middleton and colleagues showed that Egyptians were among the populations that resisted data sharing [12]. Ahram and colleagues showed that among Jordanians there has been an apparent change of opinion regarding data sharing between 2010 and 2020. Specifically, approximately 60% in 2010 thought that the involvement of non-Jordanian researchers with their biospecimens and health data would not influence their decision to participate in biobank research as only 15% thought it would have a negative influence [21]. However, in a follow-up study in 2020, over 80% of survey respondents thought that the possibility of transferring biospecimens outside the country would negatively affect their approval to give open consent for biomedical research [53].

Our findings contrast with studies from other regions demonstrating higher rates of approval of data sharing. For example, in a study exploring the public attitudes in Latvia, approximately 70% of the participants would favor sharing of biobank samples among countries in the European Union [29]. This was also evident in a study conducted in South Africa in which more than 70% of respondents agreed to share their samples with institutions in foreign countries [54]. Other studies have shown that the public appreciates the importance of sharing and its role in enhancing scientific discoveries and improving healthcare [37, 55, 56]. Although the value of data sharing may appear to be a global trend, as recently reported by the multi-national study of Middleton et al. [12], reservations seem to exist in some populations. Openness to data sharing was not found among the Swiss public, where only 11.7% of survey respondents were willing to donate their data freely [57].

We can offer several determinants that might explain the reluctance of the public to data-sharing. These include concerns with informational privacy and trust. For example, Shabani and colleagues analyzed 15 empirical studies investigating the attitudes of research participants and the public towards genomic data sharing [58]. Their results revealed a wide variety of interrelated concerns including the personal perceptions of controllability and sensitivity of data that they share with others. Additionally, Kalkman and colleagues performed a narrative review of the empirical evidence addressing patients' and public attitudes towards the use of health data for research purposes and showed that support for data sharing is conditioned on the value of the research, risk minimization, protection of privacy, data security, transparency, accountability and trust [59].

We found certain aspects to be of little importance to the Arab public, one of which included returning individual research results. In one study, many Jordanians were not in favor of receiving individual research results regardless of whether the results revealed a treatable or untreatable disease [21]. These data showing a lack of interest in return of individual research results contrast with previous studies showing that Egyptians and Jordanians desired such information [21, 22]. A further qualitative exploratory study is warranted to further determine the public beliefs and attitudes regarding individual research results.

Finally, our study showed several differences between the four countries regarding several of the investigated constructs. For example, Morocco had significantly higher scores regarding “attitudes toward trust and privacy” compared with the other countries, whereas Egypt demonstrated significantly higher scores on “attitudes toward biobank research” compared with Jordan. These results suggest that caution should be applied in generalizing results from one country in the Middle East to the other countries in the region. Nevertheless, despite the differences in the constructs shown among the countries in our study, we did not find any country-dependent significant differences in the “willingness to   participate in biobank research.”

## Limitations

We recognize several limitations of our study. First, most respondents were mainly educated individuals holding a bachelor’s degree and higher and did not include many individuals from lower educational backgrounds. Although we had previously discussed concerns with in-person recruitment coupled with face-to-face interviews that could lead to social desirability bias, our use of online recruitment methods might have given rise to selection bias toward certain social sectors. For example, online recruitment might favor individuals with higher educational levels and with better access to the internet. Such a selection bias could fail to achieve a representative sample that would limit the generalizability of our results. Another limitation in our results involves a lack of understanding of our respondents to genomics-related research as well as the risks and benefits of data sharing. Another point worth mentioning is the survey was performed at the start of the COVID-19 pandemic, which might have influenced people's attitudes given the conflicting scientific information that was given to the public at that time and the resulting negative effects on the credibility of biomedical research. Despite these limitations, the study adds a novel contribution to the literature regarding biobanks in this region and highlights  the need for further research.

## Conclusions and recommendations

Our study demonstrates challenges to enhancing participation in biobank research that include addressing inaccurate perceptions about biobanking practices and clarifying attitudes that are contrary to normative practices, Gaskell and Gottweis warned that lack of knowledge of biobanks among Europeans would lead to the end of such projects [35] and similar concerns could also be applicable to LMICs. Accordingly, we encourage awareness efforts that open a dialogue with the public that would enhance their knowledge and consider alternative attitudes toward biobanks. We further urge actively involving the public in the governance structures of the biobanks to build trust between the public and the other stakeholders involved in biobanks.

Our results uncovered several areas of concern that should be the focus of qualitative exploratory research. For example, our survey showed that the return of individual research results was of little importance to many participants, which stands in contrast to other studies investigating attitudes of the public. Additional qualitative research would help clarify potential misunderstandings and uncovered concerns that are not obtainable in quantitative surveys. Moreover, our survey showed that participants with more accurate perceptions of biobanks and with higher educational levels were less willing to participate in biobank research. These results might be explained by postulating that more accurate perceptions or higher education levels confers a greater understanding of the risks associated with biobank research (e.g., violation of privacy) or that leads one to be wary of participation. Such individuals might be less trustful of the governance structures of biobanks compared with others. Qualitative research could further explore these and other potential underlying factors.

Finally, our survey demonstrated that participants harbored concerns about international data-sharing. Such reluctance is disconcerting as collaborative efforts with global data sharing can yield novel discoveries and targeted treatment strategies for genetic and rare diseases at the global health level. A failure to participate in global data sharing would further enhance the genomic health equity gap between LMICs and HICs. However, LMICs hold legitimate concerns regarding “equity” with data sharing. For example, there might be apprehension that the sharing of data will not lead to a concurrent and equitable benefit to the researchers, participants, and the communities from where the data were collected. There are also issues regarding ownership of the data and data security. We recommend mixed-method research among the relevant stakeholders to explore their knowledge and perspectives regarding data-sharing. Additionally, research should investigate regulatory frameworks that exist at the national level in the Arab countries, whether material and data sharing agreements are negotiated merely between individual institutions and researchers, and the role of research ethics committees in a regulatory framework.

## Supplementary Information

**Additional file 1**. Research Objectives of the study and the corresponding null hypotheses.

**Additional file 2**. Final Questionnaire used in the survey study.

**Additional file 3**. Information Sheet and Informed Consent Form Presented to the Participants.

**Additional file 4**. Post Hoc multiple comparisons test.

**Additional file 5**. Bivariate analysis between demographic variables and individual question items of the construct "attitudes toward biobank research".

**Additional file 6**. Bivariate analysis between demographic variables and individual question items of the constructs.

**Additional file 7**. Correlation analysis between demographics and constructs.

**Additional file 8**. Correlation between the different constructs of each country and the willingness to participate in biobank research.

## Data Availability

The datasets during and/or analyzed during the current study are available at https://osf.io/263vu/?view_only=2a1d02a68dcd4334a800a39cd01ef51d
