# Supplementary material for: Perceptions, attitudes, and willingness of the public in low- and middle-income countries of the Arab region to participate in biobank research
Source: BMC Med Ethics. 2022 Dec 1;23:122. doi: 10.1186/s12910-022-00855-z (PMC9713115; doi:10.1186/s12910-022-00855-z)
Supplement: Supplementary file 1 — Additional file 1. Research Objectives of the study and the corresponding null hypotheses. [file 12910_2022_855_MOESM1_ESM.docx]

**Additional File 1: Research objectives of the study and the corresponding null hypotheses**

**Research Objectives**

OBJECTIVE 1:

To explore the association between perceptions, attitudes of the public toward biobanks, attitudes toward privacy and trust, and their willingness to participate in biobank research.

OBJECTIVE 2:

To determine the factors associated with the perceptions, attitudes, and the willingness of potential participants to participate in biobank research (i.e., donate biospecimens and their health-related data).

OBJECTIVE 3:

To determine differences between the countries of the Middle East regarding the willingness to participate in biobank research.

OBJECTIVE 4

To determine differences in constructs between the different countries.

**HYPOTHESIS of RESEARCH STUDY**

H1: There is no association between willingness to participate in biobank research and perceptions about sample donation, attitudes toward biobank research, and attitudes toward privacy and trust.

H 2: Demographics have no significant impact on perceptions, attitudes, and willingness to participate in biobank research

H 3: There are no significant differences in the willingness of the public to participate in biobank research between the four different countries.

H 4: The different countries do not have variations in the constructs (i.e., “perceptions” and “attitudes”).
