# Supplementary material for: Perceptions, attitudes, and willingness of the public in low- and middle-income countries of the Arab region to participate in biobank research
Source: BMC Med Ethics. 2022 Dec 1;23:122. doi: 10.1186/s12910-022-00855-z (PMC9713115; doi:10.1186/s12910-022-00855-z)
Supplement: Supplementary file 2 — Additional file 2. Final Questionnaire used in the survey study. [file 12910_2022_855_MOESM2_ESM.docx]

**Additional file 2: Final Questionnaire used in the survey study**

Survey ID#: ___________________ Date: _______ /___________/_________

Day Month Year

**Perceptions, Attitudes, and Likely Behaviors of the Public Towards Donation and Storage of Biological Specimens and Health Information for Biobanking Research**

DATA COLLECTED FROM THIS STUDY ARE CONFIDENTIAL AND WILL BE USED FOR SCIENTIFIC PURPOSES ONLY.

On the following pages, we will ask your views about taking part in biobank research. Your answers to this survey are very important. Your answers will help us understand how people think and feel about having their biological samples and health information stored, shared, and used in a biobank. This will inform how biobank research is done in the future. Thank you.

**What is this survey about?** This survey is about your views on taking part in medical research. We want to understand what you think about having your biological samples and copies of your health information being stored in a central location (often called a “biobank”) and used for medical research. Health information may include information from your medical record such as test results and information about diseases. It may also include information from your blood, tissues, and other samples, as well as genetic information (DNA). By completing this survey you will help inform how biobank research should be conducted in the future.

1. **Personal Background Information:**
2. Country ______________ Region __________________ Governorate ______________
3. Residence: urban  1 rural  2
4. Where is this survey taking place?

Public clinic/hospital  1 Private clinic  2

Non-profit clinic/hospital  3 Cancer clinic/hospital  4

1. Medical condition: Cancer  1 Other  2
2. Gender: Male  1 Female  2
3. What is your age in years? _______
4. Highest level of education completed:

1 No schooling

2 Less than primary school (up to grade 8)

3 Primary school (grade 8)

4 Vocational/technical training diploma

5 Secondary school (grade 12)

6 University degree (B.A./B.S.)

7 Postgraduate degree (M.D., Ph.D., J.D., etc.)

8 Other (please specify): __________________

1. Religion: Muslim 1 Christian 2  3 Other (please specify): __________________
2. Marital Status:

Never Married.……… 1 Married…………..…2 Divorced………..………3

Widowed………..…… 4  5Other: ­­­­­­­­­­­__________________

1. Do you have children………Yes……… 1 No…… 2
2. How religious are you?

Not at all religious.……… 1 Not very religious….… 2 Somewhat religious..……… 3

Very Religious……………  4

1. Have you ever participated in medical research? Yes……… 1 No……… 2
2. If Yes, which of the following studies have you participated in before (check all that apply)?

a. Drug Clinical trial: Yes….…. 1 No…… 2

b. Blood samples donation: Yes….. .. 1 No…… 2

c. Genetic research: Yes…....  1 No…… 2

d. Questionnaire/Interview study: Yes……  1 No…... 2

e. Do not know Yes……  1 No…... 2

1. Which of the following research would you consider volunteering for in the future (check all that apply)?

a. Drug Clinical trial: Yes  1 No  2

b. Blood sample donation: Yes  1 No  2

c. Genetic research Yes  1 No  2

d. Questionnaire/interview study: Yes  1 No  2

e. Do Not Know Yes  1 No  2

1. Have you previously heard of the term “biobank” before today?

Yes……. 1 No……  2 Not sure…….  3

| 1. **Perceptions About Biobanks: 13**   **Please state whether you believe the following statements by answering Yes, No, or Not sure** | | | |
| --- | --- | --- | --- |
|  | (3)Yes | (2)No | (1)Not sure |
| **Donation** |  |  |  |
| 1. Biobank research can lead to better medical treatments for future generations. |  |  |  |
| 1. Biobank research can lead to improvement in an individual’s health. |  |  |  |
| 1. People will have to spend monies to donate biological samples. |  |  |  |
| 1. Biobank research will only benefit private drug companies. |  |  |  |
| **Storage** |  |  |  |
| 1. People who donate their biological samples will not be able to request to have their samples destroyed in the future. |  |  |  |
| 1. Biological specimens given to a biobank can be sold to anyone. |  |  |  |
| **PRIVACY** |  |  |  |
| 1. Personal medical information stored in a biobank will remain private. |  |  |  |
| 1. Personal medical information stored in a biobank might be revealed to unauthorized people. |  |  |  |
| **Data Sharing** |  |  |  |
| 1. Biological samples can be shared with researchers in other institutions in my country |  |  |  |
| **Research** |  |  |  |
| 1. Researchers are more interested in making money from donated biological samples than doing good research. |  |  |  |
| 1. A person might be cloned if he/she donates a biological samples to a biobank. |  |  |  |
| 1. Biological samples will be used for the production of biological weapons. |  |  |  |
| **RETURN OF RESULTS** |  |  |  |
| 1. Researchers will contact people if the analysis of their biological specimens shows risk for disease. |  |  |  |

| **Aspects of biobank research that affect willingness to donate** To help determine which aspects of biobank research are important in determining whether you will want to donate a tissue sample and medical information to a biobank, we want you to think about each of the following statements and indicate how important it would mean to your decision to participate in biobank research 13 | | | | | |
| --- | --- | --- | --- | --- | --- |
|  | Very important [1] | Important [2] | Moderately Important [3] | Slightly important [4] | Not important [5] |
| **Donation** | 3 | | 2 | 1 | |
| 1. Future research on my biological samples could improve healthcare for people in the future. |  |  |  |  |  |
| 1. Future research on my biological samples will be reviewed by an ethics committee. |  |  |  |  |  |
| 1. My personal health will improve from my donation. |  |  |  |  |  |
| 1. My religion approves of my donating biological samples. |  |  |  |  |  |
| **Storage** |  |  |  |  |  |
| **PRIVACY** |  |  |  |  |  |
| 1. My medical information will remain private. |  |  |  |  |  |
| 1. if the analysis of my biological samples reveal any stigmatizing information about me, this will be kept private. |  |  |  |  |  |
| **Data Sharing** |  |  |  |  |  |
| 1. My biological samples and medical information will be shared with researchers who are from other countries. |  |  |  |  |  |
| 1. Access to my biological samples and medical information in the biobank will be strictly controlled by an oversight committee. |  |  |  |  |  |
| 1. Researchers outside of my institution will not receive any biological samples or medical information that directly identifies me. |  |  |  |  |  |
| **Return of Results** |  |  |  |  |  |
| 1. I will be able to obtain the genetic results from the analysis of my biological samples. |  |  |  |  |  |

1. **Preferences toward Types of Biobanks**

Please imagine that you have been asked to allow your biological samples and medical information to be placed in a biobank at your local hospital or healthcare organization. In addition, this particular biobank will share your biological samples and medical information with researchers at your local hospital or other healthcare organizations who wish to use the samples and medical information in the biobank for research. In addition, this particular biobank will place your medical information in a large online national database that anyone in the public can access if their research is approved by an oversight committee. This is to make it easier for researchers from other institutions in your countries and in other countries to use your medical information in research.

Below are different conditions in which a biobank can operate. We will ask you to rate how likely you would participate in each specific biobank. You may want to read all of the conditions before answering.

Condition 1: **Broad Consent and Coded Database**

1. You will give Broad Informed consent in which you will agree for your biological samples and medical information to be used for any type of medical research in the future (unspecified) without the necessity of you giving any further consent. Review of future research will be under the oversight of a research ethics committee.
2. A coded database will be used in which the biobank will remove your personal information such as name, address, national ID number, and birth date and replace it with a code (a number linked to your name) before it is it is given to other researchers. Biobank officials will have access to the code that can link back to your identity. The coding will enable the biobank to re-identify your medical information in case a result of testing that can improve your health that will be communicated to the treating physician to discuss with you. Also, being able to re-identify your information will allow for the addition of your follow-up medical information to the biobank that increases the value of the research. However, there will be a small possibility that an unauthorized person obtains the code and be able to identify your medical information.

Please answer the following question about the imaginary biobank described in Condition 1.

|  | Yes Definitely  [1] | Yes Probably  [2] | Not sure  [3] | Probably  Not  [4] | Definitely  Not  [5] |
| --- | --- | --- | --- | --- | --- |
| If you were asked, would you take part in this biobank? |  |  |  |  |  |

**Condition 2: Broad Consent and Irreversibly Anonymized Database**

1. You will give Broad Informed consent in which you will agree for your biological samples and medical information to be used for any type of medical research in the future (unspecified) without the necessity of you given any further consent. Review of future research will be under the oversight of a research ethics committee.
2. The biobank will remove permantely your personal information such as name, address, national ID number, and birth date from your samples and medical information (information will be irreversibly anonymized). Biobank managers and other researchers will not be able to identify you and hence it will not be possible to notify your doctor or you of any new results that might affect your health. Also, any future medical information will not be able to be added to the biobank, which limits the value of future research. However, the protection of your medical information is now more secured, that is, an unauthorized person will not be able to identify your medical information.

Please answer the following question about the imaginary biobank described

|  | Definitely  Yes  [1] | Probably Yes  [2] | Not sure  [3] | Probably  Not  [4] | Definitely  Not  [5] |
| --- | --- | --- | --- | --- | --- |
| If you were asked, would you take part in this biobank? |  |  |  |  |  |

**Condition 3: Tiered Consent and coded samples**

1. Instead of being asked to consent to “any” type of future research, a Tiered consent will be asked, in which you will be given a choice as to what types of medical research you will allow your biological samples and medical information to be used. For example, you can choose whether you want future research to be restricted to only your health condition, restricted to illnesses that are in the same category of your illness, or no restrictions on the types of medical research that can be performed in the future. For each choice, it will not be necessary for you given any further consent. Review of such future research will be under the oversight of an ethics committee.
2. **This type of database will be coded.**

Please answer the following question about the imaginary biobank described

|  | Definitely  Yes  [1] | Probably Yes  [2] | Not sure  [3] | Probably  Not  [4] | Definitely  Not  [5] |
| --- | --- | --- | --- | --- | --- |
| If you were asked, would you take part in this biobank? |  |  |  |  |  |

**Condition 4. Tiered Consent and Irreversibly Anonymized Database**

Please answer the following question about the imaginary biobank described

|  | Definitely  Yes  [1] | Probably Yes  [2] | Not sure  [3] | Probably  Not  [4] | Definitely  Not  [5] |
| --- | --- | --- | --- | --- | --- |
| If you were asked, would you take part in this biobank? |  |  |  |  |  |

**Condition 5: Recontact for Research Consent**

1. Instead of “broad consent” or “tiered consent” y**ou will be re-contacted for consent for ANY future research.**
2. In order to contact you, t**his type of database will be coded**

Please answer the following question about the imaginary biobank described

|  | Definitely Yes  [1] | Probably Yes  [2] | Not sure  [3] | Probably  Not  [4] | Definitely  Not  [5] |
| --- | --- | --- | --- | --- | --- |
| If you were asked, would you take part in this biobank? |  |  |  |  |  |

| 1. **Attitudes toward biobank research 11**   Please rate the extent to which you agree or disagree (1 = strongly agree to 5 =strongly disagree) with each of the following statements: | | | | | | |
| --- | --- | --- | --- | --- | --- | --- |
|  | Strongly Agree [1] | Agree [2] | No opinion[3] | Disagree [4] | Strongly Disagree [5] | I do not understand [6] |
| **Donation** |  |  |  |  |  |  |
| 1. People should donate biological samples to improve the health of future generations. |  |  |  |  |  |  |
| 1. People should donate biological samples even if there will not be a direct health benefit to them. |  |  |  |  |  |  |
| 1. People who donate biological specimens should receive financial compensation that is in addition to any travel expenses. |  |  |  |  |  |  |
| **Storage** |  |  |  |  |  |  |
| 1. If people change their minds, they should have the right to withdraw their consent for the use of their biologiocal samples. |  |  |  |  |  |  |
| **Privacy** |  |  |  |  |  |  |
| 1. Researchers must maintain the privacy of a donor’s medical information when they perform research. |  |  |  |  |  |  |
| **Data Sharing** |  |  |  |  |  |  |
| 1. It is acceptable for biological samples to be shared with researchers at other institutions in my country. |  |  |  |  |  |  |
| 1. It is acceptable for samples to be shared with researchers at other institutions |  |  |  |  |  |  |
| 1. The legal authorities should have the right to obtain my genetic results when necessary. |  |  |  |  |  |  |
| 1. Researchers should receive governmental approval prior to exporting samples out of the country. |  |  |  |  |  |  |

| **RETURN of results** |  |  |  |  |  |  |
| --- | --- | --- | --- | --- | --- | --- |
| 1. If the analysis of my biological specimens reveal a disease that can be treated or prevented, then either I or my doctor should be informed of these results. |  |  |  |  |  |  |
| 1. Even if the analysis of my biological specimens reveal a genetic disease that cannot be treated or prevented, I still want to be informed of these results. |  |  |  |  |  |  |

| 1. **Attitudes toward Privacy and Trust 3**   Please rate the extent to which you agree or disagree (1 = strongly agree to 5 =strongly disagree) with each of the following statements: | | | | | | |
| --- | --- | --- | --- | --- | --- | --- |
|  | Strongly Agree [1] | Agree [2] | No opinion[3] | Disagree [4] | Strongly Disagree [5] | I do not understand [6] |
| I worry about the privacy of my medical information when I share it with my doctor |  |  |  |  |  |  |
| I worry about the privacy of my medical information when I share it with researchers |  |  |  |  |  |  |
| I trust the individuals in charge of biobanks |  |  |  |  |  |  |

| 1. **Willingness to participate in biobank research:** Please rate the extent to which you would do each of the following: **5** | | | | | |
| --- | --- | --- | --- | --- | --- |
|  | **Definitely**  **yes [1]** | **Probably**  **Yes [2]** | **Not sure [3]** | **Probably not [4]** | **Definitely Not [5]** |
| 1. If you are asked to give your medical information for research, would you agree to do it? |  |  |  |  |  |
| 1. If you are asked to give saliva for research, would you agree to do it? |  |  |  |  |  |
| 1. If you are asked to give a urine sample for research, would you agree to do it? |  |  |  |  |  |
| 1. If you are asked to give a blood sample for research, would you agree to do it? |  |  |  |  |  |
| 1. If you are asked to participate in genetic research, would you agree to do it? |  |  |  |  |  |

**Thank you very much for taking the time to complete this survey! Your answers are very important to us and we really appreciate your time.**
