# Supplementary material for: Perceptions, attitudes, and willingness of the public in low- and middle-income countries of the Arab region to participate in biobank research
Source: BMC Med Ethics. 2022 Dec 1;23:122. doi: 10.1186/s12910-022-00855-z (PMC9713115; doi:10.1186/s12910-022-00855-z)
Supplement: Supplementary file 3 — Additional file 3. Information Sheet and Informed Consent Form Presented to the Participants. [file 12910_2022_855_MOESM3_ESM.docx]

**Additional File 3: Information Sheet and Informed Consent Form Presented to the Participants**

**Information Sheet: What is this survey about?**

This survey relates to your views on participating in medical research. We want to understand what you think about storing your samples and copies of your health information in a central location (often called a “biobank”) and using it for medical research. Health information may include information from your medical history such as test results and information about your illness. It may also include information from analyzes of your blood, tissue and other samples, as well as genetic information (about genetic material/DNA). By completing this survey, you will help to know how biobanking research can be done in the future

Why is biobanking research important? Does biobank research benefit participants?

The purpose of biobanking research is to help people in general. This research does not directly help people who participate in the biobank, but it may help them in the future.

Do biobanks share information that could identify a person with third parties?

Biobanks do not share information that could easily identify a person. Everyone in the biobank gets a code. Biobanks share this code with third parties, but remove personal information such as names, addresses, and dates of birth before health information is shared. All information that can help identify you is removed from your bio samples and health information, and therefore, the chance of identifying you from your health information is very small.

What if someone agreed to participate and later changed their mind?

People participating in a biobank can request that their health information be removed from the bank at any time, but if health information has been previously shared, it will not be possible to remove this information from ongoing studies.

Please read the following definitions before you start filling out the survey:

Biobanks: Institutions that store various biological samples such as body fluids (from blood, urine, etc.) or samples of body tissues (such as tumor samples). These biological samples are collected from healthy or sick donors in addition to all their data, or data on their health, or The samples they donated, or sometimes from their families. Researchers use these samples to find new diagnostic or therapeutic methods for various diseases, especially incurable diseases such as cancer.

DNA: The material found inside all living things like us, which transmits genetic traits from one generation to another, such as the color of the eyes, and is also responsible for the heredity of some diseases.

Genetic/hereditary disease: a condition caused by a defect in the DNA that is passed from one generation to the next.

Privacy: The right of a person to keep his information, affairs and personal relationships confidential from others.

Scientific research: is the use of systematic methods to describe, explain, or predict an observed phenomenon such as diseases and to discover treatments for these diseases.

Scientific Research Ethics Committee: A committee consisting of individuals with expertise in medicine and ethics who review the scientific and ethical aspect of research studies.

Clinical drug trial: A type of research that checks whether a drug is being tested.

A study carried out on blood samples: a type of scientific research that uses a biological sample (blood) in order to analyze many information, including DNA data.

Consent: The voluntary consent to participate in scientific research. This consent becomes “informed consent” when the research participants are informed of the details of the research in which they are participating, their rights, and the risks associated with their participation in it.

**Informed Consent**

**Title: Public percept**ions**, attitudes, and willingness regarding donation and storage of human biological samples for biobanking research.**

Introduction: The project aims to increase understanding of individuals' ideas and attitudes towards donating biological samples and storing them within research biobanks within the Arab region. So we came to you to ask if you would like to join this survey. The survey will take about 20 minutes. This study was approved by the Scientific Research Ethics Committee in December 2019

Number of Participants: You are one of about 800 people who have been asked to participate in this research. We plan to increase the number of participants from Egypt, Jordan, Sudan and Morocco.

Participation is voluntary: your participation is voluntary and you have the right to decline participation. Your refusal will not have any negative consequences for the health care services you receive.

Action: We will ask you to fill out a questionnaire. The questionnaire consists of 5 pages and is divided into the following sections that ask about a) age, marital status, education level and if you have ever participated in research; b) questions about your ideas about biobanking and donating biological samples; c) Questions about specific positions on the concept of biobanking and donation of biological samples; d) Questions about your views on the consent procedure. You may decide not to answer any question that bothers you.

Benefits: Your participation does not benefit you directly. But your participation will provide us with valuable information that will improve the conduct of medical research not only here, but in the entire Arab region.

Risks: Your participation in this study has little or no risks, as your name will not be directly associated with your answers.

Privacy and Confidentiality: To keep your answers anonymous, you will not write your name on the survey.

Right to Withdraw: You have the right to withdraw from the study at any time without giving any reasons. Your withdrawal will not have any negative consequences.

Contact information: If you have any questions or concerns about this study, you can speak with any member of the research team who contacted you to fill out this questionnaire.
