# Supplementary material for: Perceptions, attitudes, and willingness of the public in low- and middle-income countries of the Arab region to participate in biobank research
Source: BMC Med Ethics. 2022 Dec 1;23:122. doi: 10.1186/s12910-022-00855-z (PMC9713115; doi:10.1186/s12910-022-00855-z)
Supplement: Supplementary file 4 — Additional file 4. Post Hoc multiple comparisons test. [file 12910_2022_855_MOESM4_ESM.docx]

**Additional file 4: Post Hoc multiple comparisons test (Turkey's range test) for differences in countries**

|  | | **Perceptions about Biobanks** | **Aspects of biobank research that affect willingness to donate biospecimens** | **Attitudes towards activities in biobanking research** | **Attitudes towards trust and privacy** | **Willingness to participate in biobank research** |
| --- | --- | --- | --- | --- | --- | --- |
| Country | | p-value | p-value | p-value | p-value | p-value |
| Egypt | Morocco | 1.000 | 0.048* | 0.141 | 0.000* | 0.116 |
|  | Jordan | 0.328 | 0.001* | 0.003* | 0.538 | 0.062 |
|  | Sudan | 0.000* | 1.000 | 0.108 | 0.635 | 0.835 |
| Morocco | Egypt | 1.000 | 0.048* | 0.141 | 0.000* | 0.116 |
|  | Jordan | 0.658 | 0.996 | 0.970 | 0.003* | 0.994 |
|  | Sudan | 0.045* | 0.080 | 0.938 | 0.000* | 0.432 |
| Jordan | Egypt | 0.328 | 0.001* | 0.003* | 0.538 | 0.062 |
|  | Morocco | 0.658 | 0.996 | 0.970 | 0.003* | 0.994 |
|  | Sudan | 0.332 | 0.007* | 0.570 | 0.993 | 0.433 |
| Sudan | Egypt | 0.000* | 1.000 | 0.108 | 0.635 | 0.835 |
|  | Morocco | 0.045* | 0.080* | 0.938 | 0.000* | 0.432 |
|  | Jordan | 0.32 | 0.007* | 0.570 | 0.993 | 0.433 |

*Mean difference is significant at 0.01

**Table 10: Willingness to participate in Biobank research**

| **Country** | **Constructs** | | | |
| --- | --- | --- | --- | --- |
|  |  | **Perceptions about biobank** | **Attitudes towards biobank** | **Attitudes toward Privacy and Trust** |
| **EGYPT** |  | **-0.065**  **0.117**  **593** | **0.495**  **0.000**  **593** | **0.128**  **0.002**  **593** |
| **MOROCCO** |  | **-0.312**  **0.010**  **68** | **0.443**  **0.000**  **68** | **0.003**  **0.980**  **68** |
| **JORDAN** |  | **-0.179**  **0.048**  **123** | **0.394**  **0.000**  **123** | **0.052**  **0.571**  **123** |
| **SUDAN** |  | **-0.139**  **0.061**  **183** | **0.364**  **0.000**  **183** | **-0.025**  **0.733**  **183** |

**Table 10.  OLD TABLE Willingness to participate in biobank research**

| **Country** | **Constructs** | | |
| --- | --- | --- | --- |
|  | **Perceptions about sample donation** | **Attitudes toward biobank** | **Attitudes toward Privacy and Trust** |
| **EGYPT** | **-0.110**  **0.007**  **593** | **0.492**  **0.000**  **593** | 0.127  **0.000**  **593** |
| **MOROCCO** | **-0.381**  **0.001**  **68** | **0.437**  **0.000**  **68** | 0.097  **0.432**  **68** |
| **JORDAN** | **-0.214**  **0.017**  **123** | **0.473**  **0.000**  **123** | **-0.049**  **0.592**  **123** |
| **SUDAN** | **-0.329**  **0.000**  **183** | **0.452**  **0.000**  **183** | **0.013**  **0.857**  **183** |

**From table, it can be inferred that in EGYPT;**

· Willingness to participate in biobank research and perceptions about sample donation shares negative weak relationship (r=-0.065).

· Willingness to participate in biobank research and attitudes towards participation in biobanking research shows moderate positive relationship (r=0.495).

· Willingness to participate in biobank research and attitudes toward trust and privacy shows weakest positive bond (r=0.128).

**The correlation among variables in Morocco is;**

· Willingness to participate in biobank research and perceptions about sample donation shares negative moderate relationship (r=-0.312).

· Willingness to participate in biobank research and attitudes towards participation in biobanking research shows moderate positive relationship (r=0.443).

· Willingness to participate in biobank research and attitudes toward trust and privacy shows weakest positive bond (r=0.003).

**The correlation among variables in Jordan was,**

· Willingness to participate in biobank research and perceptions about sample donation shares negative weak relationship (r=-0.179).

· Willingness to participate in biobank research and attitudes towards participation in biobanking research shows moderate positive relationship (r=0.394).

· Willingness to participate in biobank research and attitudes toward trust and privacy shows weakest positive bond (r=0.052).

**The correlation in Sudan is;**

· Willingness to participate in biobank research and perceptions about sample donation shares negative weak relationship (r=-0.139).

· Willingness to participate in biobank research and attitudes towards participation in biobanking research shows moderate positive relationship (r=0.364).

· Willingness to participate in biobank research and attitudes toward trust and privacy shows weakest negative bond (r=0.025).
