# Supplementary material for: Perceptions, attitudes, and willingness of the public in low- and middle-income countries of the Arab region to participate in biobank research
Source: BMC Med Ethics. 2022 Dec 1;23:122. doi: 10.1186/s12910-022-00855-z (PMC9713115; doi:10.1186/s12910-022-00855-z)
Supplement: Supplementary file 7 — Additional file 7. Correlation analysis between demographics and constructs. [file 12910_2022_855_MOESM7_ESM.docx]

**Additional File 7: Correlation analysis between demographics and constructs**

| **VARIABLES** | **Residence** | **Medical condition** | **Gender** | **Age** | **Education** | **Religion** | **Marital Status** | **Children** | **Religiosity** |
| --- | --- | --- | --- | --- | --- | --- | --- | --- | --- |
| **Willingness to participate in biobank research** | 0.035  0.272  967 | -0.13  0.689  967 | -0.003  .919  967 | -0.047  0.142  967 | -0.076*  0.018  967 | 0.005  0.886  967 | 0.027  0.400  967 | 0.036  0.257  967 | 0.011  0.732  967 |
| **Perceptions about biobanks** | -0.025  0.435  967 | -0.022  0.499  967 | -0.008  0.801  967 | -0005  0.883  967 | - 0.034  0.294  967 | 0.031  0.331  967 | - 0.031  0.338  967 | 0.036  0.257  967 | 0.007  0.816  967 |
| **Attitudes towards biobank research** | 0.012  0.699  967 | -0.033  0.311  967 | 0.032  0.326  967 | -0.11**  0.001  967 | -0.016  0.611  967 | -0.004  0.899  967 | -0.006  0.861  967 | -0.021  0.513  967 | 0.014  0.657  967 |
| **Attitudes toward trust and privacy** | -0.098**  0.002  967 | 0.012  0.699  967 | -0.009  0.088  967 | -0.059  0.065  967 | -0.102**  0.002  967 | 0.027  0.399  967 | -0.006  0.862  967 | -0.004  0.890  967 | -0.052  0.105  967 |
