# Supplementary material for: Perceptions, attitudes, and willingness of the public in low- and middle-income countries of the Arab region to participate in biobank research
Source: BMC Med Ethics. 2022 Dec 1;23:122. doi: 10.1186/s12910-022-00855-z (PMC9713115; doi:10.1186/s12910-022-00855-z)
Supplement: Supplementary file 8 — Additional file 8. Correlation between the different constructs of each country and the willingness to participate in biobank research. [file 12910_2022_855_MOESM8_ESM.docx]

**Additional file 8: Correlation between the different constructs of each country and the willingness to participate in biobank research**

| **Country** | **Constructs** | | |
| --- | --- | --- | --- |
|  | **Perceptions about biobank research** | **Attitudes toward biobank research** | **Attitudes toward Privacy and Trust** |
| **EGYPT** | -0.110  0.007  593 | 0.492  0.000  593 | -0.127  0.000  593 |
| **MOROCCO** | -0.381  0.001  68 | 0.437  0.000  68 | -0.097  0.432  68 |
| **JORDAN** | -0.214  0.017  123 | 0.473  0.000  123 | -0.049  0.592  123 |
| **SUDAN** | -0.329  0.000  183 | 0.452  0.000  183 | -0.013  0.857  183 |
